# Occupational injuries and their sociodemographic, workplace, and behavioral determinants among large-scale factory workers in Ethiopia: a cross-sectional study

**DOI:** 10.1590/0102-311XEN162923

**Published:** 2024-08-19

**Authors:** Ana Oña, Robel Tadele Forsido, Olena Bychkovska, Andrea Aegerter, Germán Guerra, Yacob Alemu Bizuneh, Kirubel Manyazewal Mussie

**Affiliations:** 1 Schweizer Paraplegiker-Forschung, Nottwil, Switzerland.; 2 Department of Health Sciences and Medicine, University of Lucerne, Lucerne, Switzerland.; 3 School of Public Health, Africa Medical College, Addis Ababa, Ethiopia.; 4 School of Health Sciences, Zurich University of Applied Sciences, Winterthur, Switzerland.; 5 Instituto Nacional de Salud Pública, Cuernavaca, México.; 6 Université de Genève, Geneva, Switzerland.; 7 School of Medicine, Debre Markos University, Debre Markos, Ethiopia.; 8 Labour Administration, Labour Inspection and Occupational Safety and Health Branch, International Labour Organisation, Geneva, Switzerland.; 9 Addis Centre for Ethics and Priority Setting, Addis Ababa University, Addis Ababa, Ethiopia.

**Keywords:** Occupational Health, Work, Safety, Risk Assessment, Salud Laboral, Trabajo, Seguridad, Medición de Riesgo, Saúde Ocupacional, Trabalho, Segurança, Medição de Risco

## Abstract

Workplace injuries constitute a serious and growing public health concern worldwide. Despite work-related injuries being highly common, especially among workers in the manufacturing industry, their growing complexities are not adequately addressed in the current literature. Therefore this study aims to investigate the association between sociodemographic, workplace, and behavioral characteristics with work-related injuries among large-scale factory workers in Ethiopia. A cross-sectional study was conducted from February to April 2020 with 457 workers selected from large-scale factories in Addis Ababa, the capital of Ethiopia. Survey data included sociodemographic characteristics, working and safety conditions, and behavioral factors as predictors of occupational injuries. A logistic regression model was fitted to estimate the probability of injury and identify its associated factors. The 12-month prevalence of work-related injuries was 25%. Most injuries occurred at midnight (8.8%). Factors associated with work-related injury were excessive working hours (OR = 3.26; 95%CI: 1.26-8.41), cigarette smoking (OR = 2.72; 95%CI: 1.22-6.08), and manual handling (OR = 2.30; 95%CI: 1.13-4.72). Use of personal protective equipment reduced the odds of injury (OR = 0.42; 95%CI: 0.21-0.83). Although our estimated prevalence of occupational injury was lower than that found in other studies, our findings suggest that actions on modifiable conditions must be taken to reduce the burden of workplace injuries in Ethiopia. The results could inform preparedness and policy efforts aimed at improving worker safety and health.

## Abstract (es)

Los accidentes laborales constituyen un grave y creciente problema de salud pública mundial. Aunque este tipo de accidente es frecuente, especialmente entre los trabajadores de las industrias manufactureras, su creciente complejidad no ha sido abordada adecuadamente en la literatura actual. El objetivo de este estudio fue analizar la asociación entre las características sociodemográficas, ambientales y conductuales y los accidentes laborales en trabajadores de grandes fábricas de Etiopía. Se trató de un estudio transversal, realizado de febrero a abril de 2020 con 457 trabajadores de grandes fábricas en Adís Abeba, la capital de Etiopía. Los datos recopilados contenían características sociodemográficas, condiciones de trabajo, de seguridad y factores conductuales como predictores de accidentes laborales. Se utilizó un modelo de regresión logística para estimar la probabilidad de lesión e identificar sus factores asociados. La prevalencia de accidentes laborales en 12 meses fue del 25%. La mayoría de las lesiones se produjeron a medianoche (8,8%). Los factores asociados a los accidentes laborales fueron el exceso de horas de trabajo (OR = 3,26; IC95%: 1,26-8,41), el tabaquismo (OR = 2,72; IC95%: 1,22-6,08) y la manipulación manual (OR = 2,30; IC95%: 1,13-4,72). El uso de equipos de protección individual redujo la probabilidad de lesiones (OR = 0,42; IC95%: 0,21-0,83). Aunque la prevalencia estimada de accidentes laborales fue inferior a la de otros estudios, los resultados apuntan a acciones que deben adoptarse para reducir la carga de accidentes laborales en Etiopía. Los resultados permiten reflexionar sobre los esfuerzos de preparación y las políticas dirigidas a mejorar la seguridad y la salud de los trabajadores.

## Abstract (pt)

Acidentes de trabalho são preocupações de saúde pública sérias e crescentes em todo o mundo. Apesar de acidentes de trabalho serem comuns, especialmente entre trabalhadores de indústrias manufatureiras, suas crescentes complexidades não são adequadamente abordadas na literatura atual. O objetivo deste estudo foi investigar a associação de características sociodemográficas, de ambiente e comportamentais com acidentes de trabalho em trabalhadores de fábricas de grande porte na Etiópia. Um estudo transversal foi conduzido de fevereiro a abril de 2020 com 457 trabalhadores selecionados de fábricas de grande escala em Adis Abeba, capital da Etiópia. Os dados da pesquisa incluíram características sociodemográficas, condições de trabalho e segurança e fatores comportamentais como preditores de acidentes de trabalho. Ajustou-se um modelo de regressão logística para estimar a probabilidade de lesão e identificar seus fatores associados. A prevalência de 12 meses de acidentes de trabalho foi de 25%. A maioria das lesões ocorreu à meia-noite (8,8%). Os fatores associados aos acidentes de trabalho foram jornada excessiva (OR = 3,26; IC95%: 1,26-8,41), tabagismo (OR = 2,72; IC95%: 1,22-6,08) e manuseio manual (OR = 2,30; IC95%: 1,13-4,72). O uso de equipamentos de proteção individual diminuiu a chance de lesão (OR = 0,42; IC95%: 0,21-0,83). Embora nossa prevalência estimada de acidentes ocupacionais tenha sido menor em comparação com outros estudos, nossos resultados apontam para ações pendentes sobre condições modificáveis a serem tomadas para reduzir a carga de acidentes de trabalho na Etiópia. Os resultados poderão informar os esforços de preparação e políticas destinados a melhorar a segurança e a saúde dos trabalhadores.

## Introduction

Occupational safety is a serious and complex public health concern worldwide. Although occupation is generally reported to have a positive impact on health and well-being ^1^, evidence also point toward different negative effects on health, such as physical injuries ^2^ and mental health issues ^3^ ^,^ ^4^. The International Labor Organization (ILO) estimates that 2.78 million workers die annually due to workplace injuries and another 374 million are affected by non-fatal work-related accidents ^5^. Additionally, occupational injuries are a major cause of disability. In 2019, among all causes of diseases, occupational injuries accounted for 3.9% of all years lived with disability (YLD), and the main risks were ergonomics, noise, and injuries ^6^. During and after the COVID-19 pandemic, more occupation-related health problems, such as burnout, mental health issues, and injuries, were recorded among in healthcare workers ^7^ ^,^ ^8^ ^,^ ^9^ and other sectors ^10^ ^,^ ^11^. Similar to the consequences, the causes of occupational injuries are complex and result from the interaction of multiple factors at both the individual and national levels ^12^. Health status and exposure to occupational risks are strongly and differently influenced by the type of activity, working and employment conditions, and regulatory frameworks such as occupational safety and health measures and social security systems ^13^ ^,^ ^14^ ^,^ ^15^.

Occupation-related physical and mental health issues are major concerns across different employment contexts in Ethiopia ^16^ ^,^ ^17^ ^,^ ^18^ ^,^ ^19^ ^,^ ^20^ ^,^ ^21^ ^,^ ^22^. As of 2021, Ethiopia’s working-age population (15-64 years of age) was estimated to be 55.9 million ^21^, and this population group is projected to grow by two million every year over the next decade ^23^. To improve working conditions and protect the workforce, the Ethiopian government has been taking various measures, including the development of a regulation on occupational safety and health in 1945 (last revised in 2014), adopting the *ILO Convention n. 155 of 1981*, in 1991, and the *Labor Proclamation n. 377/2003* and *Labor Proclamation n. 515/2007* on public civil servants ^24^.

Despite these efforts and the rapidly growing labor force, occupational health challenges remain a significant public health concern in Ethiopia. This challenge is exacerbated by the limited availability of occupational health services in the face of a rapidly changing economic and employment context in the country ^24^. According to ILO, Ethiopia has the second highest mortality rate due to work-related injuries among 47 countries in the African region (28 per 100,000 working-age population) ^5^. In 2019, the percentage of YLD due to occupational risks in Ethiopia was 4.4%, which is 14% higher than the global level (3.84%) ^6^. A recent systematic review on work-related injuries among construction workers in Ethiopia reported a prevalence of 46.78% of work-related injury ^17^. Similarly, other studies have reported non-fatal workplace injuries, such as eye and head damage ^25^. Men are more affected than women (5.6% and 3.3%, respectively), and there are disparities within the country, ranging from 3.1% (Addis Ababa, Ethiopia) to 4.9% (Amhara, Ethiopia) of YLD ^6^.

Despite the high prevalence of work-related injuries, especially among manufacturing industry workers, most studies on this topic focus on the prevalence of occupational injuries, only cover a few specific sectors (textile, metal, among others), and focus on other parts of the country ^25^ ^,^ ^26^. Conducting this study in Addis Ababa, the largest and capital city of Ethiopia, was considered appropriate in order to obtain more representative data for other parts of the country, as the city has a diverse population of approximately five million. Therefore, this study aims to investigate the association of sociodemographic, work environment, and behavioral characteristics with work-related injuries among large-scale factory workers in Addis Ababa. The objective is to increase the number of published findings on work-related injuries in Ethiopia, while providing a source of evidence for the selected companies from which workers were sampled to implement effective interventions and cost-benefit analysis for improving prevalent health and safety measures. The study can also help governmental bodies and decision-makers from different sectors involved in developing and improving occupational safety and health policies, safety legislation, and occupational health services.

## Methods

### Study design and sample selection

This cross-sectional study was conducted among the manufacturing companies registered in Akaki, located in the south of Addis Ababa, and part of the Akaki Kality Sub-City Administration. There are six large-scale manufacturing industries (i.e., with > 150 employees each) registered by the city administration: Ethio-metal factory (267 employees), KK textile factory (207 employees), Zenit oil factory (1,316 employees), Heineken beer factory (510 employees), Gezeto industry (219 employees), and Ethio Kacha factory (440 employees), with a total of 2,959 employees. The 2-stage sampling technique was used for the sampling process: one for the factories and one for workers. Three of the six factories were selected using a simple random sampling technique to ensure representativeness. The three selected factories were Ethio-metal factory, Heineken beer factory, and Ethio Kacha factory. The number of employees was selected using simple random sampling. The allocation of employees for each selected factory was determined by proportional allocation using nws = n*(Nw/N); in which: *nws* = sample size for each selected factory; *Nw* = number of employees in each factory; *n* = total sample size; and *N* = total number of employees in the selected factories. The sample size was determined using the population proportion formula with a 95% confidence interval (95%CI). The following formula was used to calculate the sample size:

$$n=\frac{z_{\alpha /2}^{2}*p(1-p)}{d^{2}+\frac{z_{\alpha /2}^{2}*p(1-p)}{N}}=\frac{1.96^{2}*0.5(1-0.5)}{d^{2}+\frac{1.96^{2}*0.5(1-0.5)}{2,959}}=340$$

in which: *n* = required sample size; *p* = proportion of employees, assumed to be 50%; *Z* = confidence interval, which is usually set at 95% or 1.96; *d* = margin of error, usually set at 5% or 0.05; *N* = total number of employees in the selected factories.

Considering that the total number of employees in all factories was 2,959, the sample size was set at 340. Considering a non-response rate of 5% and a design effect of 1.5%, the final sample size required was 480.

### Data collection tools and procedures

Data were collected from February 23 to April 24, 2020, using a questionnaire consisting of 54 questions. Written informed consent was obtained from participants after providing an explanation of the purpose, benefits, and risks of the study and the individual’s right to choose whether or not to participate. The study participants were informed that there was no direct financial benefit or risk from the study and that the study findings would be used to develop strategies for injury prevention and control mechanisms among workers in the factories. For confidentiality purposes, the names of the respondents were not included in the questionnaire, so that none of the authors had access to information that could identify individual participants during or after data collection.

The survey questions covered various aspects of work-related injuries, such as sociodemographic, behavioral issues, use of personal protective equipment (PPE), and work environment. For the purposes of this study, work-related injuries were defined as physical harm sustained by individuals as a result of their work activities. Examples include fractures or dislocations from falls on construction sites. A pre-test was conducted before the actual data collection. During this pre-test, questions that were difficult for participants to understand were rephrased to make them more understandable. To ensure the status of occupational injuries within a one-year period, injury-related data were reviewed and documented from the records of the factory clinics.

### Outcome variable

The outcome of this study was work-related injuries. This dichotomous variable took the value of 1 if the employee reported a work accident in the past 12 months and zero if otherwise.

### Statistical analysis

A logistic regression model (univariable and multivariable) was fitted to estimate the probability of having a work injury. The regression was adjusted considering 13 potential predictors from the questionnaire, separated into three groups: sociodemographic factors; work environment; and behavioral factors. The sociodemographic and behavioral factors represent the characteristics of the workers, while the work environment represents the characteristics of the factories. A pre-test with dependent and possible independent variables was conducted to better identify the one-to-one relationships and select the covariables. From the 54 questions, only the variables that showed a significant relationship with the dependent variable and the personal characteristics as a control at a p-value < 0.25 were considered. For the first group of sociodemographic factors, the variables were gender, age, and salary. To reduce variability, logarithmic transformation was applied to the salary data. For the second group of work environment factors, we considered overwork (more than 48 hours), safety training, workplace supervision, and manual handling activities. The final group (behavioral factors) included the following variables: alcohol consumption; khat chewing; cigarette smoking; sleep disorders; job satisfaction; and use of PPE. Only questionnaires with complete information on all variables of interest were used. More details about the selected variables can be found in Supplementary Material (Box S1; https://cadernos.ensp.fiocruz.br/static//arquivo/suppl-csp-1629-23_2304.pdf).

## Results

### Sample characteristics

A total of 480 employees were invited to participate in this study, of which 457 participated. For this analysis, only the completed questionnaires were used, which reduced the sample to 396 employees. Table 1 shows the descriptive characteristics of the employees, with the main variables used in the logistic regression and divided by work-related injuries. The overall prevalence of these injuries in the sample was 25%, or 99 cases out of 396 respondents. Most employees were male (303 participants, 76.5%), and the mean age of the sample was 33.9 years. Compared with females, males had more work injuries (85% for males and 15% for females). There was no difference in salary between the two groups (no injury vs. injury). Most employees reported using PPE, with 257 (86.5%) in the no work-related injury group and 74 (74.7%) in the work-related injury group.

**Table 1:** Descriptive statistics.

Parameter/Level	No injury	Injury	p-value
	n (%)	n (%)	
Gender			
Male	219 (73.7)	84 (84.8)	0.034
Female	78 (26.3)	15 (15.2)	
Age [mean (SD)]	33.9 (6.7)	34.5 (6.1)	0.425
Salary [mean (SD)]	8.3 (0.9)	8.3 (0.8)	0.925
Use of PPE			
No	40 (13.5)	25 (25.3)	0.01
Yes	257 (86.5)	74 (74.7)	
Overwork			
No	286 (96.3)	88 (88.9)	0.011
Yes	11 (3.7)	11 (11.1)	
Regular safety supervision			
No	164 (55.2)	62 (62.6)	0.241
Yes	133 (44.8)	37 (37.4)	
Safety training			
No	164 (55.2)	62 (62.6)	0.241
Yes	133 (44.8)	37 (37.4)	
Manual handling			
No	102 (34.3)	22 (22.2)	0.033
Yes	195 (65.7)	77 (77.8)	
Smoking			
No	277 (93.3)	82 (82.8)	0.004
Yes	20 (6.7)	17 (17.2)	
Alcohol consumption			
No	109 (36.7)	39 (39.4)	0.719
Yes	188 (63.3)	60 (60.6)	
Chewing khat			
No	250 (84.2)	87 (87.9)	0.463
Yes	47 (15.8)	12 (12.1)	
Sleep disorder			
No	264 (88.9)	86 (86.9)	0.717
Yes	33 (11.1)	13 (13.1)	
Job satisfaction			
No	39 (13.1)	16 (16.2)	0.557
Yes	258 (86.9)	83 (83.8)	
Total	297	99	

PPE: personal protective equipment; SD: standard deviation.

According to the questionnaire, 94.4% of the participants worked less than 48 hours per week (286 in the no injury group, 88 in the injury group). Overwork, defined as working more than 48 hours per week, was 3.7% in the no work-related injury group and 11.1% in the work-related injury group (11 participants in both groups). Lack of regular safety supervision and safety training was reported by 226 of the participants (57.1%). The work of 272 employees (68.7%) involved manual handling activities such as pulling, pushing, carrying, and lifting. In terms of behavior, 37 of the participants smoked cigarettes (9.3%), 248 drank alcohol (62.6%), and 59 chewed khat (14.9%). About 12% of the respondents had sleep disorders, mainly due to working in the evening, working more than 8 hours a day, and excessive heat. In both groups, more than 85% of employees reported being satisfied with their current job or their daily tasks. Table 1 details these results. More details on the other variables included in the questionnaire and on the whole sample can be found in Supplementary Material (https://cadernos.ensp.fiocruz.br/static//arquivo/suppl-csp-1629-23_2304.pdf), which contains three tables: Table S1, on sociodemographic characteristics; Table S2, on the use of PPE and work environment characteristics; and Table S3, on the patterns of work-related injuries.

Regarding the patterns of work-related injuries, most accidents occurred at night, were machines-related, and lacked the use of PPE. The most common types of injuries were cuts, abrasions, and lacerations. The main reasons for not using PPE were uncomfortable PPE (39, 59%) and lack of PPE (14, 21.2%). In terms of the work environment, 52.2% of the machines were not guarded and the weight handled was mostly medium (6kg-25kg) and heavy (25kg-50kg).

### Model analysis

**Figure 1:**
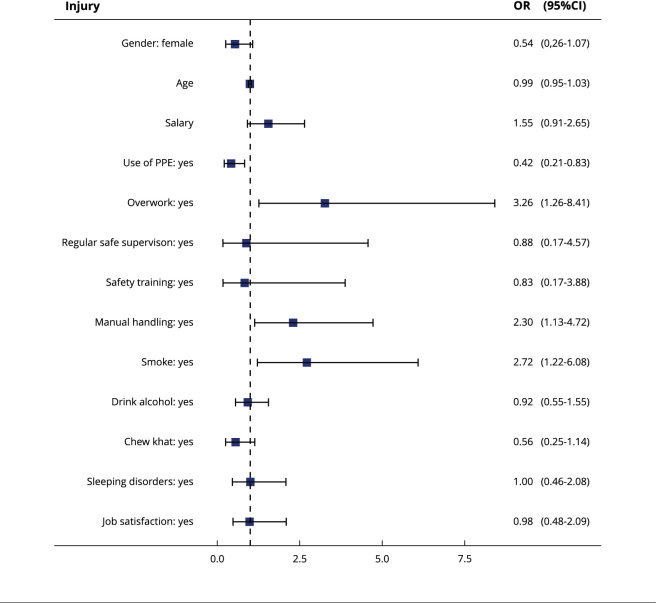
Odds ratios of the multivariable model.

Table 2 describes the variables used in the logistic regression model and the odd ratios for the univariable and multivariable logistic regression models. The results show that excessive working hours, PPE use, manual handling, and smoking were statistically significantly associated with work-related injuries. Regarding the work environment, working more than 48 hours per week or performing overtime and manual activities were associated with higher odds of suffering work-related injury, with odds ratios (OR) of 3.26 (95%CI: 1.26-8.41) and 2.3 (95%CI: 1.13-4.72), respectively. In terms of behavioral variables, smokers were 2.72 (95%CI: 1.22-6.08) times more likely to be injured at work than non-smokers. On the other hand, safety behaviors such as the use of PPE reduced the probability of having work-related injuries (OR = 0.42; 95%CI: 0.21-0.83). Figure 1 shows the odds ratios of all variables used in the model.

**Table 2:** Results of the predictors of work-related injuries among large-scale factory workers in Akaki Sub-City Addis Ababa, Ethiopia, 2020.

Parameter/Level	No injury	Injury	OR (95%CI) [univariable]	OR (95%CI) [multivariable]
	n (%)	n (%)		
Gender				
Male	219 (72.3)	84 (27.7)		
Female	78 (83.9)	15 (16.1)	0.50 (0.26-0.90)	0.54 (0.26-1.07)
Age [mean (SD)]	33.9 (6.7)	34.5 (6.1)	1.01 (0.98-1.05)	0.99 (0.95-1.03)
Salary [mean (SD)]	8.3 (0.9)	8.3 (0.8)	1.01 (0.79-1.30)	1.55 (0.91-2.65)
Use of PPE				
No	40 (61.5)	25 (38.5)		
Yes	257 (77.6)	74 (22.4)	0.46 (0.26-0.82)	0.42 (0.21-0.83)
Overwork				
No	286 (76.5)	88 (23.5)		
Yes	11 (50.0)	11 (50.0)	3.25 (1.35-7.84)	3.26 (1.26-8.41)
Regular safety supervision				
No	164 (72.6)	62 (27.4)		
Yes	133 (78.2)	37 (21.8)	0.74 (0.46-1.17)	0.88 (0.17-4.57)
Safety training				
No	164 (72.6)	62 (27.4)		
Yes	133 (78.2)	37 (21.8)	0.74 (0.46-1.17)	0.83 (0.17-3.88)
Manual handling				
No	102 (82.3)	22 (17.7)		
Yes	195 (71.7)	77 (28.3)	1.83 (1.09-3.17)	2.30 (1.13-4.72)
Smoking				
No	277 (77.2)	82 (22.8)		
Yes	20 (54.1)	17 (45.9)	2.87 (1.42-5.74)	2.72 (1.22-6.08)
Alcohol consumption				
No	109 (73.6)	39 (26.4)		
Yes	188 (75.8)	60 (24.2)	0.89 (0.56-1.43)	0.92 (0.55-1.55)
Chewing khat				
No	250 (74.2)	87 (25.8)		
Yes	47 (79.7)	12 (20.3)	0.73 (0.36-1.41)	0.56 (0.25-1.14)
Sleep disorder				
No	264 (75.4)	86 (24.6)		
Yes	33 (71.7)	13 (28.3)	1.21 (0.59-2.35)	1.00 (0.46-2.08)
Job satisfaction				
No	39 (70.9)	16 (29.1)		
Yes	258 (75.7)	83 (24.3)	0.78 (0.42-1.51)	0.98 (0.48-2.09)

95%CI: 95% confidence interval; OR: odds ratio; PPE: personal protective equipment; SD: standard deviation.

95%CI: 95% confidence interval; OR: odds ratio; PPE: personal protective equipment.

## Discussion

This study found that the overall annual prevalence of work-related injuries was 25%. Male workers were more affected than female workers. Injuries were more likely to occur at night and by machinery, and the most common reason was the lack of PPE use. Notably, many machines were unguarded and unprotected, and PPE was often not used because it was uncomfortable (Supplementary Material - Table S3; https://cadernos.ensp.fiocruz.br/static//arquivo/suppl-csp-1629-23_2304.pdf). Variables that were statistically significant associated with the outcome variable were cigarette smoking, working overtime, and manual activities. Conversely, the use of PPE was the only factor that was statistically significant associated with the prevention of occupational injuries.

Our results are consistent with those of previous studies and show that work-related injuries are a significant problem in Ethiopia ^22^ ^,^ ^27^. According to a recent pooled prevalence analysis with international comparison conducted in Ethiopia, the country’s prevalence rate of work-related injuries (25%) is relatively higher than that of China (8.3%) ^28^ and India (22.3%) ^29^ and lower than that of Malaysia (60%) ^30^, Uganda (32%) ^31^, and Nigeria (32.2%) ^32^. On the one hand, the variation in the prevalence of work-related injuries could be related to different levels of compliance with health and safety regulations (e.g., mandatory workplace supervision or maintenance of machinery).

On the other hand, the variation in prevalence rates may also be related to different injury exposure risks associated with socioeconomic factors such as the living condition of the workforce employed at the formal or informal labor market and poor working conditions, including the lack of environmental, behavioral, and safety measures. For example, lack of health and safety training and awareness or workplace supervision, limited use of PPE, and long working hours may all contribute to the occurrence of work-related injuries ^33^ ^,^ ^34^ ^,^ ^35^. This hypothesis was confirmed by our model, which showed that the odds of suffering an injury were lower if PPE was always worn (OR = 0.42; 95%CI: 0.21-0.83). This finding is in line with those of a study conducted in Nairobi, Kenya ^36^. The similarity of the findings may be because the workers who did not use PPE were not provided safety equipment by the employers, were negligent in using PPE, or felt uncomfortable using PPE in their working conditions. It could also be explained by the fact that proper use of PPE greatly reduces the occurrence of unexpected injuries in large-scale factories.

However, to focus solely on this personal preventive measure and to ignore the role of collective measures in preventing injuries or eliminating risks would limit the scope of the discussion on the findings of this study.

Individual measures, such as PPE, are typically given the least importance in hierarchical systems ^37^ of prevention and control of workplace hazards. Thus, they are considered the last “line of defense” against risks ^38^ and should be promoted together with collective measures. There is strong evidence that collective interventions are the most effective in preventing occupational injuries, especially when they do not involve individual compliance decisions ^39^.

Although not statistically significant, our results suggest that safety training and regular supervision provided mild protection against injuries (Table 2; Figure 1). Given that these collective activities are critical employer responsibilities, in addition to providing and encouraging the use of PPE, further efforts should be made to ensure that these workers receive adequate occupational safety training and health and safety supervision. This, in turn, would increase the currently low percentage of trained and supervised workers (37.9% and 37.6%, respectively; Supplementary Material - Table S2; https://cadernos.ensp.fiocruz.br/static//arquivo/suppl-csp-1629-23_2304.pdf) and prevent potential injuries.

Additionally, specific collective measures for machine operators should be prioritized. In our study, one third of all reported injuries were caused by machines, and almost 60% of the machine operators were working on an unguarded machine (Supplementary Material - Table S2; https://cadernos.ensp.fiocruz.br/static//arquivo/suppl-csp-1629-23_2304.pdf). These results highlight the need to promote engineering control measures on machines, in addition to adequate supervision and training.

Other results of this study revealed that the risk of work-related injury was higher among employees who worked more than 48 hours than their counterparts. The odds of having an injury were 3.26 times higher in employees working overtime, after adjusting for all factors (OR = 3.26; 95%CI: 1.62-8.41). This may be due to lack of concentration, lack of sleep, and substance abuse associated with long working hours. Moreover, this study showed that manual handling of objects was statistically significantly associated with prominent work-related injuries. Workers whose jobs involved manual activities were more likely to be injured than those whose jobs did not involve manual activities. This is consistent with a study conducted in Canada ^40^ and another in the rural context of Ethiopia ^41^. The higher incidence of injuries among manual workers may be due to activities with heavy or bulky loads, which involve repetitive and forceful exertion, bending, stretching, and awkward postures. Another significant finding of this study was that employees who smoked cigarettes were more likely to be injured than their counterparts. This is consistent with a study conducted in South Korea ^42^, which found that smokers were at a higher risk of suffering work-related injuries. This may be because, among other factors, smoking can lead to many other medical conditions that also interfere with the normal physical functioning of the body ^43^ ^,^ ^44^.

Our findings have broad policy and practice implications. First, they shed light on specific occupational safety and health needs that should be addressed in the surveyed population and could be extended to other companies in Addis Ababa with similar economic activities. In order to reduce workplace injuries, collective prevention measures should be actively promoted to enhance the effectiveness of individual-oriented requirements, such as the use of PPE. This should be prioritized especially among manual handling workers and machine operators, who are more likely to suffer from workplace injuries and concurrent conditions as a consequence of occupational lesions. Further attention should also be given to identifying the barriers that may hinder the implementation of such measures. Although our study describes some of the individual reasons for not using PPE (e.g., uncomfortable equipment), care should be taken not to blame these employees, but rather to look for possible organizational barriers that may limit the availability of protective equipment to all personnel (12.9% of participants reported not being provided with PPE, and those who did not use PPE cited lack of availability as the second reason [21.2%], led by discomfort [59%] as the first reason; Supplementary Material - Table S2; https://cadernos.ensp.fiocruz.br/static//arquivo/suppl-csp-1629-23_2304.pdf). A joint risk assessment (employers and employees) to find future solutions could be beneficial not only for protecting workers’ occupational safety and health and preventing injuries, but also for avoiding loss of productivity, reputation, and financial resources that these events entail for the company ^45^.

On a broader scale, compliance with these occupational safety and health measures, particularly in large-scale factories employing manual handling workers, could arguably contribute to reducing the existing burden of disability in Ethiopia. There is also a need to improve working conditions among workers in Addis Ababa by promoting healthy lifestyles. Occupational safety and health measures could also include mandatory smoke-free areas at the workplace or restrictions on smoking based on national and global regulations, such as the WHO Framework Convention on Tobacco Control ^46^.

Our findings also reinforce the need to advance the promotion of occupational safety and health policies by implementing occupational health services, especially for large-scale industry workers, such as those included in this study. In the context of economic growth, global market expansion, industrial development, and demographic dividend, the Ethiopian workforce must enjoy the best possible occupational health, which can only begin with adequate promotion of safe workplaces. This means that strong occupational safety and health policies can be based on and adapted from existing frameworks such as ILO’s C187 and C161, as well as national regulations.

We acknowledge four limitations in our study. The first is the use of self-reported data, which raises the possibility of underreporting. Injury data from other studies were mainly obtained from official company records. A second limitation relates to the 12-month recall of injury events, which may have led to recall bias. An additional limitation is that participants may have provided socially desirable responses, as some questions targeted their behavior and lifestyle practices. Lastly, as mentioned in the *Methods* section, prevalence was calculated for the respondents who completed the survey (n = 396). Statistical modeling was also conducted based on the completed questionnaires. Since the prevalence including all cases (complete and incomplete) is higher (25.6%; Supplementary Material - Table S3; https://cadernos.ensp.fiocruz.br/static//arquivo/suppl-csp-1629-23_2304.pdf), we acknowledge a slight underestimation of the reported prevalence.

## Conclusions

This study shows that work-related injuries can be strongly influenced by a variety of factors, ranging from those in the workplace environment to wider socioeconomic determinants. We found disparities in workplace injury rates by activity, with manual handling workers appearing to be at greater risk of this type of injury. Future research should focus on investigating whether these disparities are consistent across the Ethiopian labor market. This could be achieved by stratifying workplace injuries by occupational activity or worker type to identify gradients in occupational health. Further research could also use mixed methods (quantitative and qualitative) and include the perspectives of employers and occupational safety and health policy makers to fully understand the phenomenon. Efforts are needed to create a safer work environment in large-scale factory settings and beyond in Ethiopia. These could include promoting the use of protective measures and training targeted to large-scale factory workers, prioritizing manual handling workers. Greater awareness on prioritizing collective protective measures should also be promoted among factory authorities and decision-makers to address work-related risks among their employees. Moreover, our results imply the need to strengthen and implement current occupational safety and health policies and programs in Ethiopia, mainly targeting large-scale factory employees.
