# Supplementary material for: Occupational injuries and their sociodemographic, workplace, and behavioral determinants among large-scale factory workers in Ethiopia: a cross-sectional study
Source: Cad Saude Publica. 2024 Aug 19;40(8):e00162923. doi: 10.1590/0102-311XEN162923 (PMC11338599; doi:10.1590/0102-311XEN162923)
Supplement: Supplementary file 1 [file 1678-4464-csp-40-08-EN162923-s.pdf]

Supplementary Material

Box S1 Operational definitions of variables.

**Work-related injury:** Is an injury or illness caused, contributed or significantly aggravated by events or exposures in the work environment
**Use of personal protection equipment (PPE):** Specialized clothing or equipment (such as goggles, gloves, ear plug, masks, helmets, face shield, boots, protective clothing) worn by employees for protection against related health and safety hazards at the time of the survey. Personal protective equipment is designed to protect many parts of the body, such as eyes, head, face, hands, feet, and ears
**Overwork:** Do you work over 48 hours per week (8 hrs. per day)? yes, no
**Regular safe supervision:** Regular supervision done by health and safety professionals/supervisors who received short-term training on health and safety
**Safety training:** Short-term training given on health and safety to factory workers
**Manual handling:** Does your work involve manual handling activity (pulling, pushing, carrying, and lifting)? yes, no
**Smoke:** The practice of smoking cigarettes regularly (daily or occasionally)? yes, no
**Drink alcohol:** Consumption of any kind of alcohol at least two times per week
**Chew Khat:** Chewing chat leaves at least once per week
**Sleeping disorder:** The presence of sleeping problems when the worker is at work in the factory
**Job satisfaction:** Are you satisfied with your job or task required to do at Present? yes, no

Table S1 Socio-demographic characteristics .

Variables/Levels	n (%)
Gender	
Male	335 (73.3)
Female	122 (26.7)
Age (years)	
18-24	29 (6.3)
25-34	234 (51.2)
35-44	157 (34.4)
45-54	36 (7.9)
65+	1 (0.2)
Marital status	
Married	169 (36.9)
Unmarried	288 (63.0)
Religion	
Christian	369 (80.7)
Islam	88 (19.3)
Education	
No education	416 (91.0)
Have education	41 (8.9)
Residence type	
Urban	421 (92.1)
Rural	36 (7.9)
Employment pattern	
Permanent	419 (91.7)
Contract	38 (8.3)
Work experience (months)	
≤ 60	221 (48.4)
> 60	236 (51.6)
Working section	
Metal production	81 (17.7)
Fiber production	153 (22.5)
Beer production	116 (25.4)
Scrap production	9 (2.0)
Machinist	8 (1.8)
Manual machine	13 (2.8)
Mechanic	13 (2.8)
Warehouse	37 (8.1)
Forklift	15 (3.2)
Maintainer	10 (2.2)
Forman	2 (0.4)
Salary level (USD)	
≤ 5,250	269 (58.9)
> 5,250	188 (41.1)

Table S2 Use of personal protection equipment (PPE) and work environment characteristics.

Variable/Levels	n (%)
**PPE usage**	
Do you have PPE	
No	59 (12.9)
Yes	398 (87.1)
Reason for not having PPE	
PPE not provided	59 (12.9)
PPE use	
No	67 (14.7)
Yes	332 (72.6)
Reason for not using PPE	
Careless and ignorance	9 (13.6)
PPE shortage	14 (21.2)
PPE not comfortable	39 (59.0)
Lack of awareness	3 (4.6)
No PPE enforcement	1 (1.5)
PPE supply	
Workplace	329 (99.1)
Worker	3 (0.9)
**Work environment**	
Weekly working hours	
Under 48	431 (94.3)
Over 48	26 (5.7)
Health and safety supervision	
No	285 (62.4)
Yes	172 (37.6)
Job safety training	
No	284 (62.1)
Yes	173 (37.9)
Manual handling	
No	125 (27.4)
Yes	332 (72.6)
Average weight handled	
Not > 5kg	9 (2.0)
Medium 6-25kg	145 (31.7)
Heavy 25-50kg	101 (22.1)
Very heavy > 50kg	78 (17.1)
Visual concentration	
No	395 (86.4)
Yes	62 (13.6)
Vibrating tools usage	
No	408 (89.3)
Yes	49 (10.7)
Daily use of vibrating tools (hours)	
< 1	28 (6.1)
2-4	7 (1.5)
> 4	15 (3.3)
Guarded machines	
No	239 (52.3)
Yes	168 (36.8)
Maintained machines	
No	47 (10.3)
Yes	360 (78.8)

Table S3 Patterns of work-related injuries .

Variable/Levels	n (%)
Accidents in the last 12 months	
No	336 (73.5)
Yes	121 (26.5)
Number of accidents	
1	66 (14.4)
2-4	50 (10.9)
5 +	5 (1.1)
Number of sick leave days	
0	35 (7.5)
1-2	1 (0.2)
3-5	37 (8.1)
6-10	15 (3.3)
> 10	33 (7.2)
Accidents in the last 2 weeks	
No	440 (96.3)
Yes	17 (3.7)
Reason for accident	
New to the work process	1 (0.2)
Thinking about private affairs	7 (1.5)
Accident was beyond control	24 (5.3)
Work behavior	13 (2.8)
Occupational safety failure	29 (6.4)
Did not have PPE	6 (1.3)
Did not use PPE	32 (7.0)
Poor PPE	3 (0.7)
Workload	6 (1.3)
Time of injury	
Morning	21 (4.6)
Afternoon	24 (5.3)
Evening	27 (5.9)
Midnight	40 (8.8)
No information	9 (2.0)
Source of accident	
Machine	43 (9.4)
Broken glass	8 (1.8)
Fallen object	24 (5.3)
Workload/repetitive job	12 (2.6)
Sleep disorder	4 (0.9)
Slippery/Wet floor	9 (2.0)
Collusion	7 (1.5)
Type of injury	
Cuts, abrasions, lacerations	87 (19.0)
Dislocation	12 (2.6)
Sprain	10 (2.2)

PPE: personal protection equipment.
